# Effective removal of a large, mobile right atrial thrombus with the ŌNŌ retrieval device: a novel case report

**DOI:** 10.1093/ehjcr/ytaf314

**Published:** 2025-07-17

**Authors:** Victoria Y Kim, W Wyatt Lindsey, Swati Agarwal, Kinjal Parikh, Catherine E Tomasulo

**Affiliations:** Division of Paediatric Cardiology, Inova L.J. Murphy Children’s Hospital, 3300 Gallows Rd, Falls Church, VA 22042, USA; Division of Paediatric Critical Care, Inova L.J. Murphy Children’s Hospital, 3300 Gallows Rd, Falls Church, VA 22042, USA; Division of Paediatric Cardiology, Inova L.J. Murphy Children’s Hospital, 3300 Gallows Rd, Falls Church, VA 22042, USA; Alabama College of Osteopathic Medicine, Division of Clinical Sciences, 445 Health Sciences Blvd, Dothan, AL, 36303, USA; Division of Paediatric Critical Care, Inova L.J. Murphy Children’s Hospital, 3300 Gallows Rd, Falls Church, VA 22042, USA; Division of Paediatric Cardiology, Inova L.J. Murphy Children’s Hospital, 3300 Gallows Rd, Falls Church, VA 22042, USA; Division of Paediatric Cardiology, Inova L.J. Murphy Children’s Hospital, 3300 Gallows Rd, Falls Church, VA 22042, USA

**Keywords:** Right atrial thrombus, Thrombectomy, Interventional cardiology, Percutaneous removal, Anticoagulation, Case report

## Abstract

**Background:**

Large right atrial thrombi represent medical emergencies due to their potential for fatal embolization. Treatment options vary based on thrombus etiology and morphology, and include: (i) anticoagulation alone, (ii) a combination of anticoagulation and thrombolysis, (iii) transcatheter thrombectomy, and (iv) surgical thrombectomy. Here, we report the youngest and smallest patient to date to successfully undergo right atrial thrombectomy using the novel ŌNŌ transcatheter retrieval system (ŌNŌCOR LLC).

**Case summary:**

An 8-year-old male with Hodgkin's lymphoma was diagnosed with a new large, mobile thrombus associated with a port-a-cath in the right atrium on routine pre-chemotherapy echocardiogram. He was otherwise asymptomatic with a structurally normal heart. Thrombolysis with alteplase was initially attempted but was unsuccessful due to anaphylaxis. The patient subsequently underwent a partial thrombectomy of the mobile portion of the thrombus using the ŌNŌ retrieval device, successfully reducing the risk of catastrophic embolization. Follow-up echocardiography at twelve weeks and seven months confirmed further reduction in thrombus size and calcification of the remnant.

**Discussion:**

This patient presented a unique therapeutic dilemma due to several factors, including hypermobile thrombus of substantial size, recent oncologic diagnosis, and anaphylaxis to alteplase. The novel ŌNŌ was effective in encapsulating the entire thrombus, securing fragments, and thereby reducing risk of embolization during retrieval. This approach also minimized blood loss and avoided the complications associated with invasive cardiothoracic surgery in a patient with underlying malignancy.

Learning points

- The novel ŌNŌ transcatheter retrieval system is effective for performing thrombectomy, even in smaller paediatric patients.
- Partial thrombectomy followed by systemic anticoagulation is an effective approach to reduce the risk of complications associated with cardiothoracic surgery.

## Introduction

Large right atrial (RA) thrombi represent medical emergencies due to their potential for fatal embolisation. Treatment options vary based on thrombus aetiology and morphology, and include: (i) anticoagulation alone, (ii) a combination of anticoagulation and thrombolysis, (iii) transcatheter thrombectomy, and (iv) surgical thrombectomy. Given the significant morbidity and mortality associated with each approach, careful assessment of each patient’s unique risk factors is essential. Here, we report the youngest and smallest patient to date to successfully undergo RA thrombectomy using the novel ŌNŌ transcatheter retrieval system (ŌNŌCOR LLC). This case highlights the effectiveness of the ŌNŌ device in performing a partial RA thrombectomy, significantly reducing the possibility of embolisation in a high-risk scenario.

## Summary figure

**Figure:**
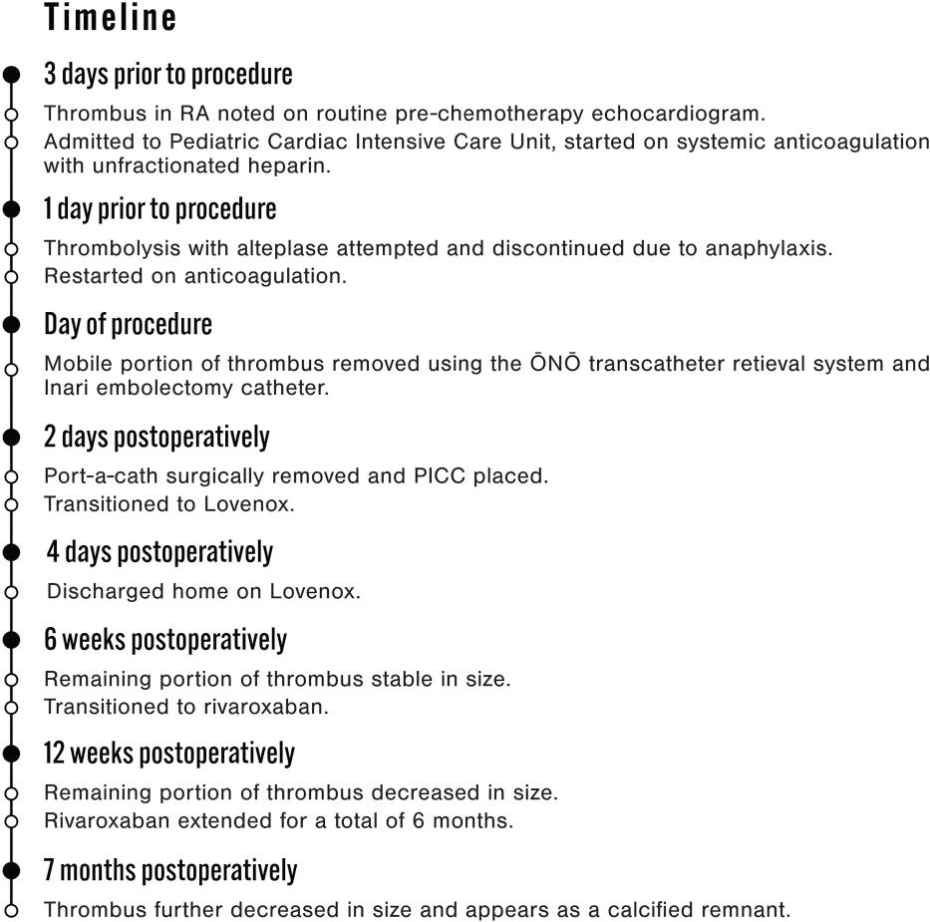


## Case presentation

An 8-year-old, 24.2-kg male with Hodgkin’s lymphoma was found to have a large, mobile RA thrombus associated with a port-a-cath on routine pre-chemotherapy echocardiogram (see Supplementary material online, *Figure S1*; *Videos S2* and *S3*). Given the risk of catastrophic pulmonary embolisation, he was emergently admitted to the paediatric cardiac intensive care unit and started on systemic anticoagulation with heparin. Upon admission, he was asymptomatic with an unremarkable physical exam. ECG showed normal sinus rhythm and prominent mid-precordial voltages; echocardiogram showed preserved cardiac function. Initial laboratory workup revealed mild anaemia with a haemoglobin of 9.5 g/dL (normal range 10.6–13.3 g/dL) and haematocrit of 29.3% (normal range 32.3%–39.7%). Otherwise, platelet count [363 × 10^3^/μL, (normal range 203–368 × 10^3^/μL)], electrolytes, and blood clotting studies were within normal limits. Bilateral upper and lower extremity doppler ultrasounds were negative for deep venous thrombosis. Per hospital protocol, he was given a bolus of heparin at 75 units/kg, followed by continuous infusion of heparin at 20 units/kg/h. Based on anti-Xa levels, heparin was adjusted to a maximum of 33 units/kg/h to maintain a goal anti-Xa level of 0.3–0.5 units/mL.

Considering the patient’s stable condition, thrombolysis with alteplase 0.6 mg/kg/h was attempted. Unfortunately, five minutes after initiation of alteplase, the patient complained of a ‘scratchy’ throat and subsequently developed hives, wheezing, and emesis, consistent with anaphylaxis. The patient was switched back to heparin. Following further discussion with a multidisciplinary team, the decision was made to proceed with thrombectomy using the ŌNŌ retrieval device on hospital day 3. Invasive surgical thrombectomy was deferred due to the patient’s oncologic diagnosis and the risks of delaying life-saving chemotherapy and of complications associated with cardiothoracic surgery.

**Figure 1:**
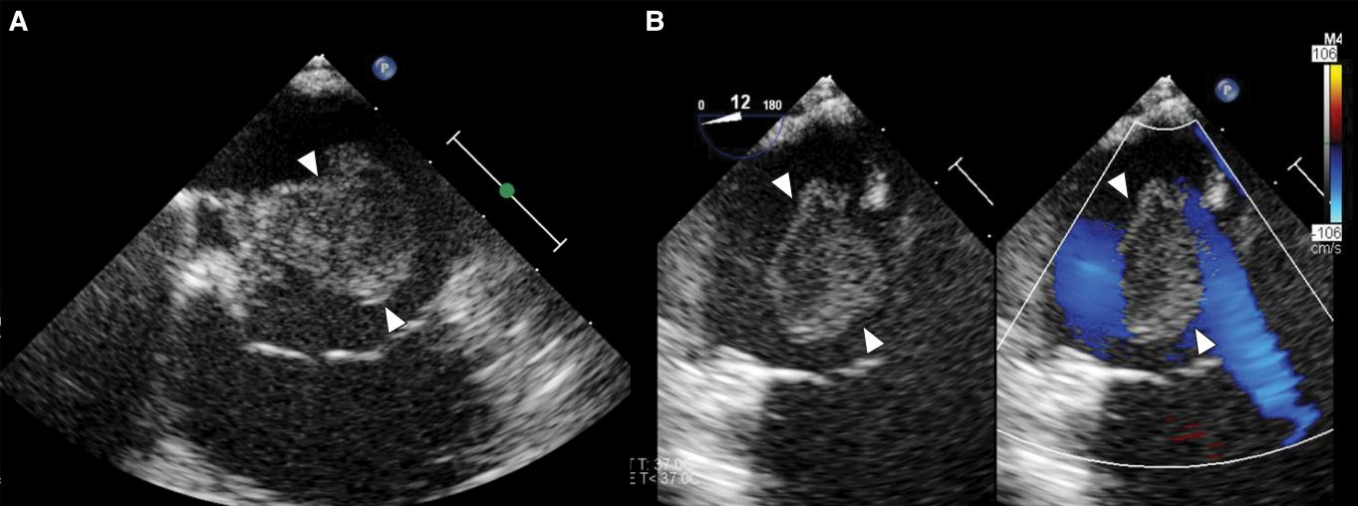
(*A*) 2D TEE: RA thrombus (white arrows) adhered to the eustachian valve. (*B*) 2D TEE colour compare: RA thrombus (white arrows) without impingement or flow disturbance of the tricuspid valve.

**Figure 2:**
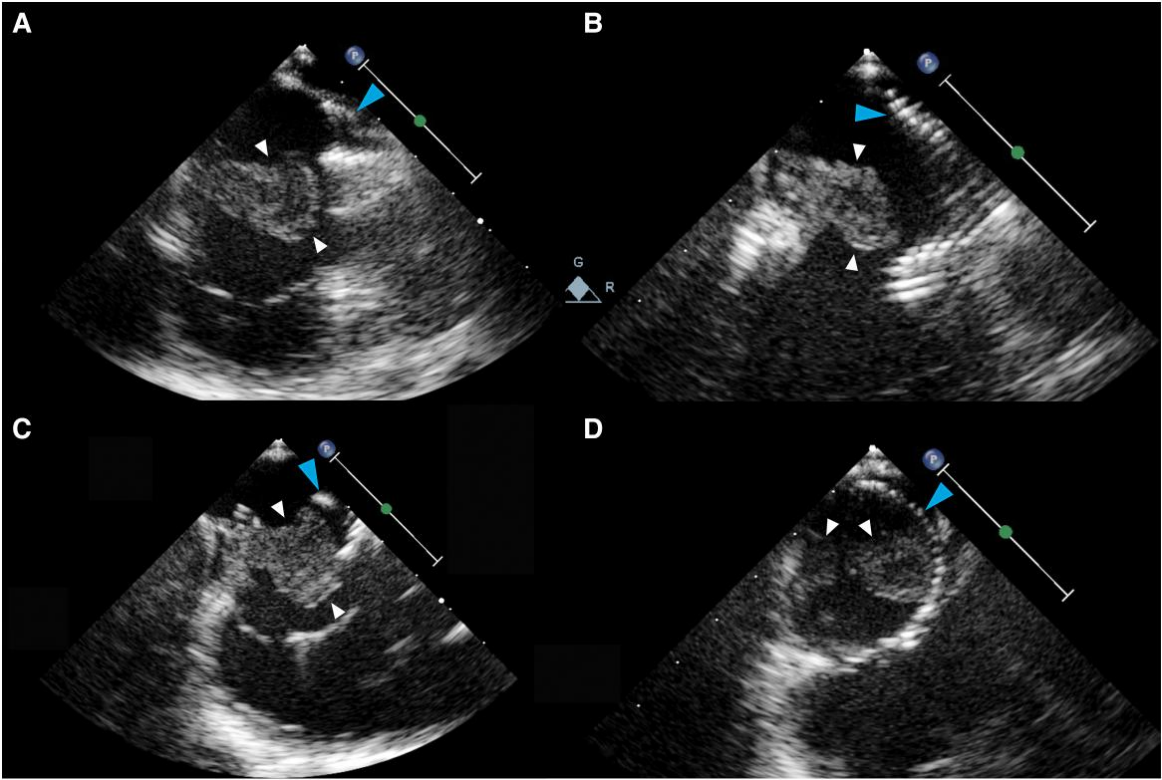
(*A*) 2D TEE: ŌNŌ device (blue arrow) positioned superior to the RA thrombus (white arrows), prior to engulfment of the thrombus. (*B*) 2D TEE: ŌNŌ device (blue arrow) fully expanded and oriented to engulf the RA thrombus (white arrows). (*C*) 2D TEE: ŌNŌ device (blue arrow) actively engulfing the RA thrombus (white arrows). (*D*) 2D TEE: ŌNŌ device (blue arrow) en face with thrombus (white arrow) within device and a portion of the thrombus (white arrow) adhered to the Eustachian valve.

**Figure 3:**
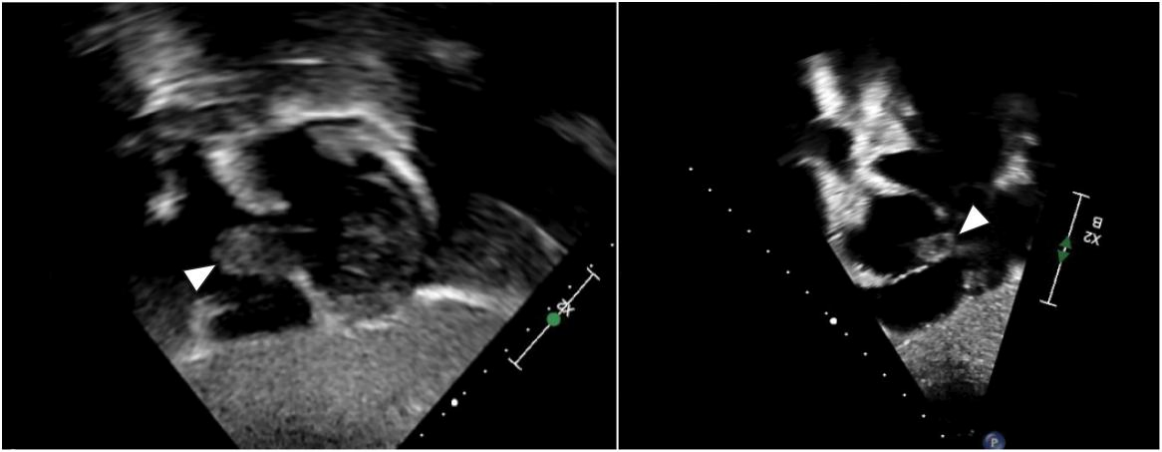
2D TEE subcostal view of pre- and post-intervention comparison of the RA thrombus (white arrows) size.

**Figure 4:**
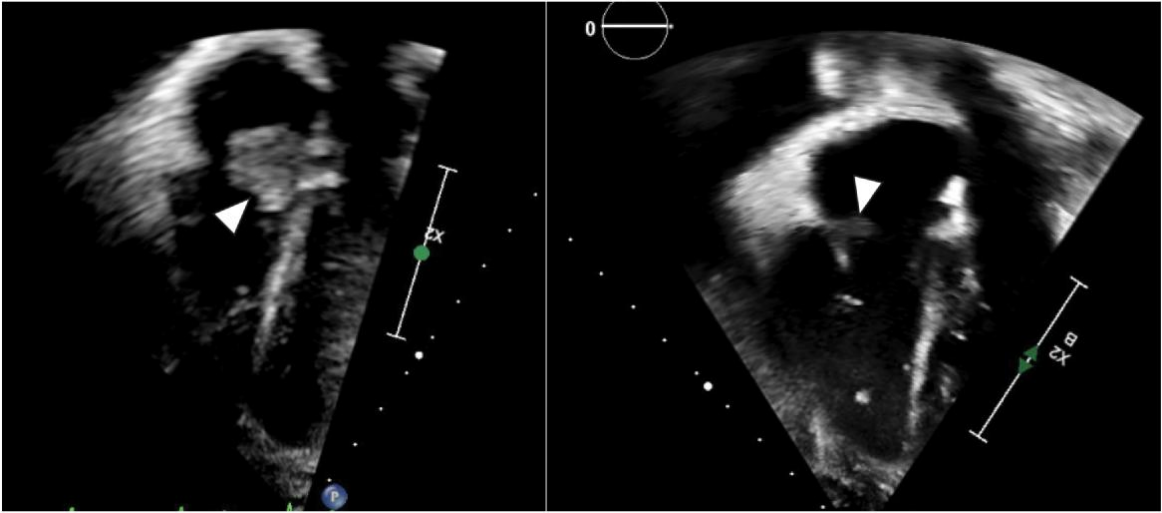
2D TEE apical view of pre- and post-intervention comparison of the RA thrombus (white arrows) size.

In the catheterisation lab, transoesophageal echocardiogram (TEE) revealed a large, mobile, echogenic mass (18 × 25 mm) attached to the RA free wall by a narrow stalk, exceeding the size of both the tricuspid valve annulus (23 mm) and main pulmonary artery (19 mm). There was no evidence of impingement or flow disturbance to the systemic veins, atrial septum, coronary sinus, or tricuspid valve (*Figure 1*, Supplementary material online, *Video S4*). A 14Fr DrySeal sheath (Gore) within a 20Fr DrySeal sheath was placed in the right internal jugular vein and positioned within the RA. The ŌNŌ retrieval system was then advanced through the 14Fr DrySeal sheath and partially opened in the RA (see Supplementary material online, *Figure S6*, *Video S5*). A 35-mm loop gooseneck snare was threaded through an MPA guide catheter, then both were advanced through the ŌNŌ. Using TEE guidance, the snare was positioned near the base of the thrombus and tightened, allowing subsequent advancement of the ŌNŌ over the snared thrombus and controlled removal of the mobile portion of the thrombus (*Figure 2*, Supplementary material online, *Video S7*). Inspection of the removed thrombus revealed an organized, fibrinous structure. A Triever20 embolectomy catheter (Inari Medical) was advanced through the DrySeal sheath to aspirate the residual, less-organized portion of the thrombus (see Supplementary material online, *Video S8*). A significantly smaller portion of the thrombus remained (*Figures 3 and 4*) and, under tension with a Raptor grasping device, was revealed to be strongly adherent to the Eustachian valve. This remaining portion was considered unlikely to embolize and, therefore, amenable to medical management.

Two days post-thrombectomy, the port-a-cath was surgically removed, and the patient was transitioned from a heparin drip to subcutaneous enoxaparin 30 mg (1.2 mg/kg/dose) every 12 h. He was discharged home four days after the procedure. At the 6-week follow-up, echocardiography showed a stable-appearing thrombus, and the patient was transitioned to rivaroxaban 5 mg (0.2 mg/kg/dose) every 12 h. Echocardiography at the 12-week follow-up demonstrated further reduction in thrombus size with a more echogenic appearance, suggesting calcification, and without any new thrombi (see Supplementary material online, *Video S9*). By 7-months post-procedure, the thrombus was smaller in size and appeared as a calcified remnant. The patient completed 6.5 months of anticoagulation, of which 5 months were with rivaroxaban.

## Discussion

This case describes an 8-year-old boy with Hodgkin’s lymphoma found to have a large, mobile RA thrombus detected on routine echocardiography, prompting urgent anticoagulation and thrombectomy with the ŌNŌ retrieval system after failed thrombolysis due to anaphylaxis. Post-procedure, he was transitioned from heparin to enoxaparin, and later rivaroxaban, with follow-up imaging showing thrombus reduction and calcification. He completed 6.5 months of anticoagulation without recurrence. The differential diagnosis included a thrombus, vegetation and cardiac tumour. Thrombus was most likely given the presence of an indwelling line and incidental finding in a clinically-well patient.

Evidence-based guidelines for managing RA thrombi, particularly in paediatric populations, are limited. The 2018 American Society of Haematology paediatric venous thromboembolism management guidelines recommend anticoagulation alone in most cases of paediatric RA thrombi, advising against thrombolysis or surgical thrombectomy. However, concerning factors such as haemodynamic status, thrombus size, and mobility, all present in this patient, may necessitate more aggressive treatment.^1^ Additionally, there is little guidance in defining high-risk thrombi by size and mobility, or selecting alternative therapies to anticoagulation.

This patient presented with a hypermobile thrombus of substantial size, posing a significant risk of complete pulmonary artery obstruction. Additional high-risk factors included a recent oncologic diagnosis, associated prothrombotic state, frequent blood transfusions, risks associated with delaying chemotherapy, and an increased risk of complications following cardiac surgery. Retrospective analyses in adults with right heart thromboemboli show lower mortality with thrombolysis compared with anticoagulation alone or surgical embolectomy (13.7% vs. 37.1% vs. 18.3%;^2^ 11.3% vs. 28.6% vs. 23.8%.^3^). Given this data and the patient’s high-risk status, thrombolysis was chosen. However, due to anaphylaxis, an alternative treatment plan was needed. In a study comparing adults with and without malignancy undergoing cardiac surgery, those with malignancy experienced significantly higher rates of red blood cell transfusions (79.5% vs. 49%, *P* < 0.0001; 88% in those with haematologic malignancies), reintubation (8.4% vs. 0.9%, *P* = 0.0009), sepsis (8.4% vs. 0.9%, *P* = 0.018), and anticoagulation-related complications (7.2% vs. 0%, *P* = 0.008).^4^ As a result, surgical thrombectomy was deferred in favour of percutaneous intervention using the novel ŌNŌ retrieval device, which had previously demonstrated favourable outcomes in a 19-year-old with a RA thrombus.^5^

Compared with traditional devices like the AlphaVac (AngioDynamics) or AngioVac (Vortex Medical) systems, the ŌNŌ device was ideal for our patient for several reasons. Rather than relying on suction mechanisms, the ŌNŌ device’s retrieval basket encapsulates the entire thrombus and secures fragments, thereby reducing the risk of embolisation during thrombus removal. Thus, the ŌNŌ device’s design was ideal for removal of this large, hypermobile, and more organized thrombus. Additionally, the ŌNŌ device's smaller sheath size was preferable to the larger sheaths required by other systems, such as the AngioVac, to accommodate large thrombi. Finally, traditional suction methods may result in significant blood loss, which would have been less ideal for our patient with underlying malignancy.^6^

Although complete thrombus removal was not achieved, partial thrombectomy significantly reduced the risk of catastrophic embolisation while avoiding the long-term complications of invasive cardiothoracic surgery. Follow-up echocardiography confirmed a reduction in the size of the remaining thrombus following 12 weeks of medical management. A total of 6 months of anticoagulation was prescribed. Long-term anticoagulant selection was guided by the 2022 European Society of Cardiology Guidelines on Cardio-Oncology, which recommended enoxaparin and non-vitamin K antagonist oral anticoagulants—specifically apixaban, edoxaban, and rivaroxaban—for cancer patients with thromboembolism and no additional bleeding risks.^7^ With this in mind, treatment was transitioned from enoxaparin to rivaroxaban due to the extended treatment duration and ease of administration.

Right heart thrombi present a complex medical challenge, particularly in paediatric patients. Management strategies must consider thrombus aetiology, morphology, and other patient-specific factors. In this case, the use of the ŌNŌ retrieval device for partial thrombectomy was critical in reducing the risk of embolisation for a high-risk thrombus. To our knowledge, this is the smallest and youngest patient to undergo catheterisation with the ŌNŌ device, highlighting its effectiveness in managing RA thrombi in paediatric populations.

## Supplementary Material

ytaf314_Supplementary_Data

## Data Availability

All relevant data for this article are included in this article and online Supplementary Data. No additional datasets were generated or analyzed.

## Lead author biography

Dr. Victoria Kim completed her paediatric residency at Inova L.J. Murphy Children's Hospital. She is currently a chief resident with plans for paediatric critical care fellowship.
