# Supplementary figures and images for: Effective removal of a large, mobile right atrial thrombus with the ŌNŌ retrieval device: a novel case report
Source: Eur Heart J Case Rep. 2025 Jul 17;9(7):ytaf314. doi: 10.1093/ehjcr/ytaf314 (PMC12291059; doi:10.1093/ehjcr/ytaf314)

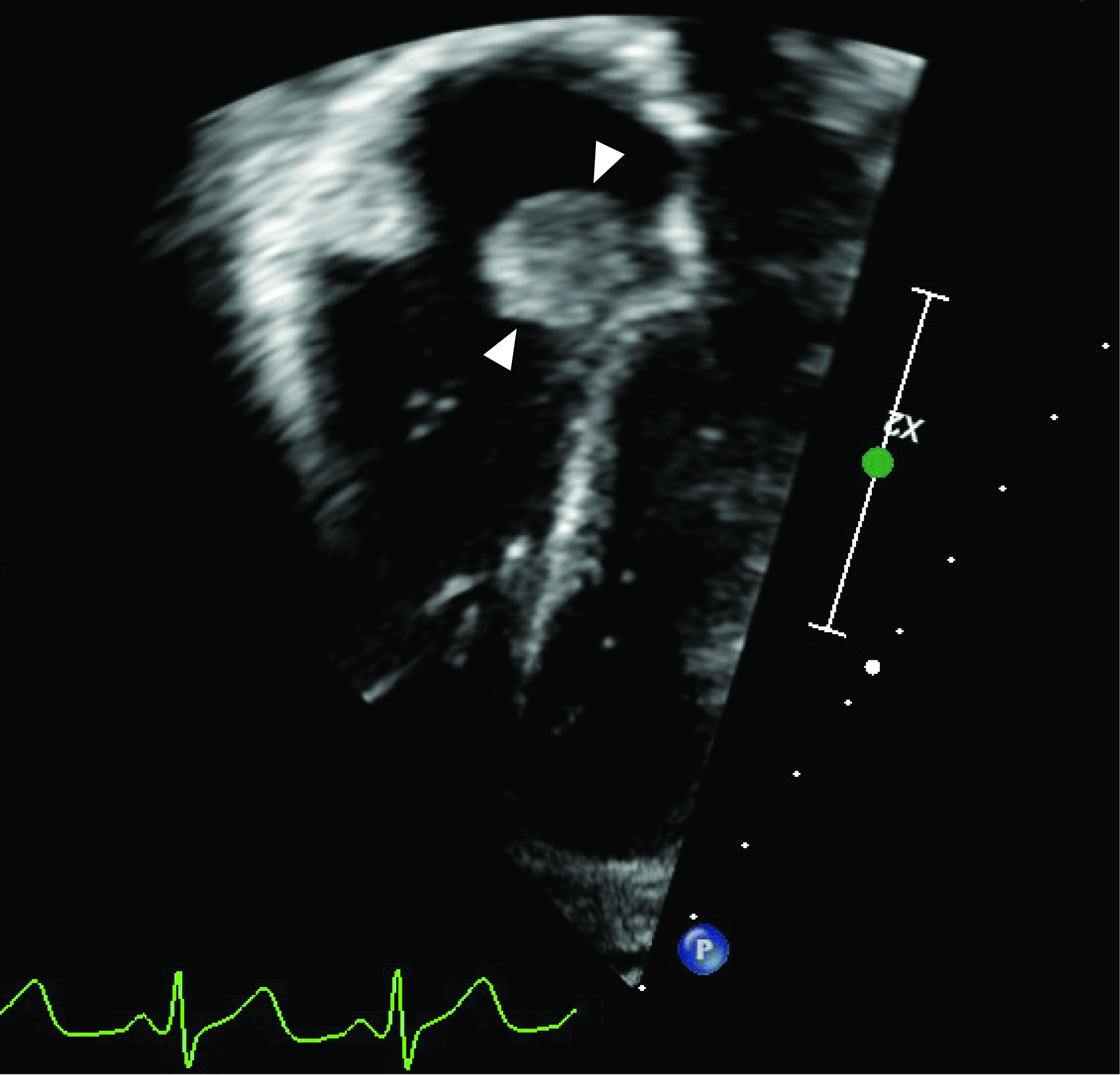

Supplement: ytaf314_Supplementary_Data [file ytaf314_supplementary_data.zip › Supplementary Figure S1.tif]
